# The signaling pathway of *Campylobacter jejuni*-induced Cdc42 activation: Role of fibronectin, integrin beta1, tyrosine kinases and guanine exchange factor Vav2

**DOI:** 10.1186/1478-811X-9-32

**Published:** 2011-12-28

**Authors:** Malgorzata Krause-Gruszczynska, Manja Boehm, Manfred Rohde, Nicole Tegtmeyer, Seiichiro Takahashi, Laszlo Buday, Omar A Oyarzabal, Steffen Backert

**Affiliations:** 1 From the School for Biomedical and Biomolecular Science, University College Dublin, Belfield Campus, Dublin-4, Ireland; 2 the Department of Microbiology, Otto von Guericke University, Leipziger Str. 44, D-39120 Magdeburg, Germany; 3 the Department of Medical Microbiology, Helmholtz Center for Infection Research, Inhoffen Str. 7, D-38124 Braunschweig, Germany; 4 the Department of Molecular Medicine, Max-Planck-Institute for Biochemistry, Martinsried, Germany; 5 the Institute of Enzymology, Biological Research Center, Hungarian Academy of Sciences, Budapest, Hungary; 6 the Department of Biological Sciences, Alabama State University, Montgomery, AL 36104, USA

**Keywords:** Rho family GTPases, Cdc42, EGF receptor, PDGF receptor, Vav2, PI3-kinase, molecular pathogenesis, cellular invasion, signaling, virulence

## Abstract

**Background:**

Host cell invasion by the foodborne pathogen *Campylobacter jejuni *is considered as one of the primary reasons of gut tissue damage, however, mechanisms and key factors involved in this process are widely unclear. It was reported that small Rho GTPases, including Cdc42, are activated and play a role during invasion, but the involved signaling cascades remained unknown. Here we utilised knockout cell lines derived from fibronectin^-/-^, integrin-beta1^-/-^, focal adhesion kinase (FAK)^-/- ^and Src/Yes/Fyn^-/- ^deficient mice, and wild-type control cells, to investigate *C. jejuni*-induced mechanisms leading to Cdc42 activation and bacterial uptake.

**Results:**

Using high-resolution scanning electron microscopy, GTPase pulldowns, G-Lisa and gentamicin protection assays we found that each studied host factor is necessary for induction of Cdc42-GTP and efficient invasion. Interestingly, filopodia formation and associated membrane dynamics linked to invasion were only seen during infection of wild-type but not in knockout cells. Infection of cells stably expressing integrin-beta1 variants with well-known defects in fibronectin fibril formation or FAK signaling also exhibited severe deficiencies in Cdc42 activation and bacterial invasion. We further demonstrated that infection of wild-type cells induces increasing amounts of phosphorylated FAK and growth factor receptors (EGFR and PDGFR) during the course of infection, correlating with accumulating Cdc42-GTP levels and *C. jejuni *invasion over time. In studies using pharmacological inhibitors, silencing RNA (siRNA) and dominant-negative expression constructs, EGFR, PDGFR and PI3-kinase appeared to represent other crucial components upstream of Cdc42 and invasion. siRNA and the use of Vav1/2^-/- ^knockout cells further showed that the guanine exchange factor Vav2 is required for Cdc42 activation and maximal bacterial invasion. Overexpression of certain mutant constructs indicated that Vav2 is a linker molecule between Cdc42 and activated EGFR/PDGFR/PI3-kinase. Using *C. jejuni *mutant strains we further demonstrated that the fibronectin-binding protein CadF and intact flagella are involved in Cdc42-GTP induction, indicating that the bacteria may directly target the fibronectin/integrin complex for inducing signaling leading to its host cell entry.

**Conclusion:**

Collectively, our findings led us propose that *C. jejuni *infection triggers a novel fibronectin→integrin-beta1→FAK/Src→EGFR/PDGFR→PI3-kinase→Vav2 signaling cascade, which plays a crucial role for Cdc42 GTPase activity associated with filopodia formation and enhances bacterial invasion.

## Background

Food-borne infections with pathogenic bacteria represent one of the leading causes of morbidity and death in humans. Estimations by the World Health Organization WHO suggest that the human population worldwide suffers from about 4.5 billion incidences of diarrhoea every year, causing approximately 1.8 million deaths [1]. *Campylobacter *has been recognized as the leading cause of enteric bacterial infection worldwide [2,3]. Two *Campylobacter *species, *C. jejuni *and to less extent *C. coli*, are most frequently found in infected persons. *Campylobacter jejuni *is a classical zoonotic pathogen, as it is part of the normal intestinal flora in various birds and mammals. Because *C. jejuni *is also present in many agriculturally important animals, it can contaminate the final products during food processing [4]. After ingestion by humans, bacteria remain motile, colonize the mucus layer in the ileum and colon, interfere with normal functions in the gastrointestinal tract, and lead to diseases associated with fever, malaise, abdominal pain and watery diarrhoea, often containing blood cells [2,3]. In addition, individuals exposed to *C. jejuni *may develop late complications, including Reiter's reactive arthritis as well as the Guillain-Barrè or Miller-Fisher syndromes [5]. Increasing amounts of data accumulated in the last decade suggest that *C. jejuni *perturbs the normal absorptive capacity of the human intestine by damaging epithelial cell functions, either directly by cell invasion and/or the production of virulence factors, or indirectly by triggering inflammatory responses [3,6-8].

It has been proposed that invasion of host cells during infection is a main source of *C. jejuni*-driven tissue damage in the intestine. Examination of intestinal biopsies from infected patients and infection of cultured human intestinal epithelial cells *in vitro *indicated that *C*. *jejuni *is capable of invading gut tissue cells [9-11]. In general, bacterial entry into host cells *in vitro *may proceed by microtubule-dependent and/or actin-dependent pathways [10,12,13]. *C. jejuni *encodes numerous outer-membrane proteins with proposed roles in bacterial adhesion such as CadF, FlpA, JlpA and PEB1 [14-17]. For example, CadF is a well-known bacterial outer-membrane protein which binds *in vitro *to fibronectin, an important extracellular matrix (ECM) protein and bridging factor to the integrin receptors [15,17-19]. INT-407 intestinal epithelial cells infected with *C. jejuni *exhibited membrane ruffling associated with bacterial entry [20]. Maximal adherence and invasion of INT-407 cells requires CadF and is accompanied with increased levels of tyrosine phosphorylation of some yet unknown host cell proteins [13,21], as well as paxillin, an integrin-associated scaffold protein [22]. However, the functional importance of these findings for host cell entry and which integrin maybe involved in this signaling remained unclear. CadF and FlpA also seems to be involved in the activation of the small Rho GTPases Rac1 and Cdc42, which are required for the cell entry [17,20], but the exact mechanisms are not yet clear. In addition, mutation of certain genes in the flagellar export system, deletion of *ciaB *(*Campylobacter *invasion antigen B), *waaF *and *kpsS *genes, led to reduced adhesion and invasion of *C. jejuni in vitro*, indicating that their corresponding proteins may also have functions in host cell invasion [23-28]. It should be noted, however, that some of these findings are not reproducible by other research groups. For example, the role of the described CiaB in invasion as well as the role of the flagellum as a potential device for the secretion of virulence factors was called into question [29]. Thus, it is not clear if the function of the flagellum during invasion is due to the secretion of bacterial factors into the medium or bacterial mobility.

Based on pharmacological inhibitor experiments, it was also reported that multiple host protein kinases, such as phosphatidylinositol 3-kinase (PI3-K), epidermal growth factor receptor (EGFR), platelet-derived growth factor receptor (PDGFR) and heterotrimeric G proteins may also play a role in epithelial cell invasion by *C. jejuni *[11,21,30]. Moreover, caveolae structures may also play a role in the invasion process because expression of dominant-negative mutants of caveolin-1 significantly decreased *C. jejuni *uptake [30]. Once internalized into epithelial cells, *C. jejuni *co-localize with microtubules [12] and survive for considerable time and consequently may induce cytotoxic responses *in vitro *[31-33]. The *C. jejuni*-containing intracellular vacuole deviates from the canonical endocytic pathway, and by inhibition of their entry into lysosomes, the bacteria may avoid elimination by the host immune system [30]. However, the molecular signaling pathways of early host cell invasion events and the complex crosstalk between bacterial and cellular factors are still widely unclear. Here we identified the signaling cascade of *C. jejuni*-induced Cdc42 activation and its role in host cell entry. We utilised a unique set of mouse knockout cell lines, GTPase pulldowns, gentamicin protection assays and high-resolution scanning electron microscopy. Our studies show the important functions of fibronectin, integrin-β1, several kinases and the guanine exchange factor Vav2 in the activation of Cdc42, and the induction of filopodia and membrane dynamics during *C. jejuni *infection. Using *C. jejuni *mutants strains we also demonstrate that the fibronectin-binding protein CadF and the flagellum may play roles in these early invasion-related signaling events.

## Methods

### Bacterial strains

The *C. jejuni *strains 81-176, 84-25 and F38011 were used in this study. The isogenic F38011Δ*cadF*, 81-176Δ*flaA/B *and 81-176Δ*flhA *mutants were kindly provided by Michael Konkel [34] and Patricia Guerry [35]. All *C. jejuni *strains were grown on *Campylobacter *blood-free selective Agar Base (Oxoid) containing *Campylobacter *growth supplement (Oxoid) or on Mueller-Hinton (MH) agar amended with 50 μg/ml kanamycin or 30 μg/ml or chloramphenicol at 37°C under microaerobic conditions (generated by CampyGen, Oxoid) for 48 hours.

### Knockout fibroblasts and other cell lines

Several mouse fibroblast knockout cell lines were cultured in RPMI1640 or DMEM medium, supplemented with 10% fetal calf serum (Gibco). Generation of the floxed FN^+/+ ^mouse fibroblast cells and FN^-/- ^knockout cells has been described elsewhere [36,37]. The FN^-/- ^cells were grown in DMEM supplemented with 10% FCS, or alternatively in serum replacement medium (Sigma Aldrich). Monolayers of GD25 mouse fibroblasts (integrin-β1^-/-^) or GD25 cells stably transfected with wt integrin β1A (GD25β1A) or several mutants (GD25β1A-TT788/89AA and GD25β1A-Y783/795F) were grown in 10% fetal bovine serum [38-40]. Mouse knockout cells deficient in focal adhesion kinase (FAK^-/- ^cells) or fibroblasts derived from c-src^-/-^, c-yes^-/-^, and c-fyn^-/- ^triple knockout mouse embryos (SYF cells) as well as stable expression of wt FAK in FAK^-/- ^cells or wt c-Src in SYF cells have been already described [41,42]. Mouse embryonic fibroblasts from Vav1/2^-/- ^deficient were prepared as described recently [43]. These cells were grown on gelatine-coated culture dishes in DMEM containing 10% FCS, non-essential amino acids and sodium pyruvate [43]. Human embryonic intestinal epithelial cells (INT-407), obtained from the American Type Culture Collection (ATCC CCL-6), were grown in MEM medium containing L-glutamine and Earle's salts (Gibco). After reaching about 70% confluency, the cells were washed two times with PBS, and then starved for 12 hours before infection.

### Infection studies

For the infection experiments, the different cell lines were seeded to give 4 × 10^5 ^cells in 12-well tissue culture plates. The culture medium was replaced with fresh medium without antibiotics 1 hour before infection. Bacteria were suspended in phosphate-buffered saline (PBS, pH 7.4), added to the cells at a multiplicity of infection (MOI) of 100, and co-incubated with host cells for the indicated periods of time per experiment.

### Inhibitor studies

The pharmacological inhibitors methyl-beta cyclodextrin (MβCD, Sigma, 1 mM-10 mM), PF-573228 (Tocris; 10 μM), AG1478 (10 μM) [44], AG370 (10 μM) [44], or wortmannin (1 μM) [21,45] were added 30 min prior to infection and kept throughout the entire duration of the experiment. Control cells were treated with the same amount of corresponding solvent for the same length of time. We have carefully checked the viability of cells in every experiment to exclude toxic effects resulting in loss of host cells from the monolayers. The experiments were repeated at least three times.

### Plasmid DNA Transfection

Eukaryotic expression vectors for human, wt PDGFRβ, dominant-negative PDGFRβ, wt EGFR and dominant-negative EGFR, were kindly provided by Drs. T. Hunter and G. Gill (University of California, USA). Myc-tagged wt Vav2 and dominant-negative Vav2 were described [43]. GFP-fusion proteins of Vav2 include wt, Vav2 Y172/159F, Vav2 R425C, Vav2 W673R and Vav2 G693R [46]. Transfection of plasmid constructs into host cells was performed using GeneJammer transfection reagent according to manufacturer's instructions (Stratagene). After 48 hours, transfected INT-407 cells were infected with C. jejuni strains for 6 hours. The efficiency of transfection was verified both by immunofluorescence microscopy and Western blotting using respective antibodies.

### siRNA Transfection

siRNA directed against human DOCK180, Vav2 and siRNA containing a scrambled control sequence were purchased from Santa Cruz. siRNA against human Cdc42 was synthesised as 5'-TTCAGCAATGCAGACAATTAA-3'. For down-regulation of Tiam-1, the siRNAs from Santa Cruz and another one obtained from MWG-Biotech (5'-ACAGCTTCAGAAGCCTG AC-3') were used simultaneously. Transfection of siRNAs was performed using siRNA transfection reagent (Santa Cruz).

### Gentamicin protection assay

After infection, eukaryotic cells were washed three times with 1 ml of pre-warmed MEM medium per well to remove non-adherent bacteria. To determine the CFU corresponding to intracellular bacteria, the INT-407 cell monolayers were treated with 250 μg/ml gentamicin (Sigma) at 37°C for 2 hours, washed three times with medium, and then incubated with 1 ml of 0.1% (w/v) saponin (Sigma) in PBS at 37°C for 15 min. The treated monolayers were resuspended thoroughly, diluted, and plated on MH agar. To determine the total CFU of host-associated bacteria, the infected monolayers were incubated with 1 ml of 0.1% (w/v) saponin in PBS at 37°C for 15 min without prior treatment with gentamicin. The resulting suspensions were diluted and plated as described above. For each strain, the level of bacterial adhesion and uptake was determined by calculating the number of CFU. In control experiments, 250 μg/ml gentamicin killed all extracellular bacteria (data not shown). All experiments were performed in triplicates.

### Cdc42-GTP pulldown assay

Cdc42 activation in infected cells was determined with the Cdc42 activation assay kit (Cytoskeleton, Inc, City, Country), based on a pulldown assay using the Cdc42-Rac1 interactive binding domain of PAK1 fused to glutathione S-transferase(GST-CRIB), also called GST-CRIB pulldown [47]. Briefly, host cells were grown to 70% confluency and serum-starved overnight. Subsequently, cells were incubated in PBS as a control or infected with *C. jejuni *(MOI of 100) in a time course. Uninfected and infected host cells were washed with PBS, resuspended in the assay buffer of the kit, and detached from dishes with a cell scraper. For a positive and negative control, a portion of the uninfected cell lysate was mixed with GTPγ-S and GDP, respectively, for 15 min. Cell lysates (treated with bacteria, GTPγ-S, GDP or untreated) were mixed with the PAK-RBD slurry (1 hour, 4°C). Finally, the beads were collected by centrifugation and washed three times with assay buffer. Activated Cdc42 was then visualized by immunoblotting as described below. To confirm equal amounts of protein for each sample, aliquots of the lysates from different time points were also analyzed by immunoblotting. The GTPase activities were quantified as band intensities representing the relative amount of active Cdc42-GTP using the Lumi-Imager F1 software program (Roche).

### G-Lisa assay

Cdc42 activation in infected cells was also determined with the G-LISA™Rac1- activation assay (Cytoskeleton). Host cells were grown to 70% confluency in tissue culture petri dishes and serum depleted overnight. The cells were infected with *C. jejuni *for the indicated times per experiment. Subsequently, cells were washed with PBS, resuspended in lysis buffer of the kit and harvested from the dishes with cell scraper. Total protein concentration in each lysate was determined by protein assay reagent of the kit. The G-LISA's contains a Rac1-GTP-binding protein immobilised on provided microplates. Bound active Cdc42 was detected with a specific antibody and luminescence. The luminescence signal was quantified by using a microplate reader (SpectraFluor Plus, Tecan).

### SDS-PAGE and immunoblotting

Proteins from transfected and/or infected host cells were separated on 10-15% polyacrylamide gels and blotted onto polyvinylidene difluoride (PVDF) membranes (Immobilon-P; Millipore). Staining with primary antibodies against FAK-PY-397 (Biomol), EGFR-PY-845, PDGFR-PY-754 (both NEB), FAK, Cdc42, RhoA, Fibronectin, integrin-β1, Tiam-1, DOCK180 or GAPDH (all Santa Cruz) was performed according to the manufacturer's instructions. As secondary antibodies, horseradish peroxidase-conjugated α-mouse, α-rabbit or α-goat IgG (DAKO) was used. Immuno-reactive bands were visualized by ECL plus Western Blotting Detection System (Amersham Biosciences). Relative FAK, EGFR and PDGFR kinase activities were quantified as band intensities of the corresponding activation-specific phospho-antibody signals related to its non-phospho control blots using the Lumi-Imager F1 software program (Roche). The strongest seen phospho-band levels per experiment were taken as 100% kinase activity.

### FESEM (Field Emission Scanning Electron Microscopy)

Host cell monolayers grown on coverslips were infected with *C. jejuni *strains for either 4 or 6 hours, then fixed with cacodylate buffer (0.1 M cacodylate, 0.01 M CaCl_2_, 0.01 M MgCl_2_, 0.09 M sucrose; pH6.9) containing 5% formaldehyde and 2% glutaraldehyde, and subsequently washed several times with cacodylate buffer. Samples were dehydrated with a graded series of acetone (10, 30, 50, 70, 90 and 100%) on ice for 15 min for each step. Samples in the 100% acetone step were allowed to reach room temperature before another change of 100% acetone. Samples were then subjected to critical-point drying with liquid CO_2 _(CPD030, Bal-Tec, Liechtenstein). Dried samples were covered with a 10 nm thick gold film by sputter coating (SCD040, Bal-Tec, Liechtenstein) before examination in a field emission scanning electron microscope (Zeiss DSM-982-Gemini) using the Everhart Thornley SE detector and the inlens detector in a 50:50 ratio at an acceleration voltage of 5 kV.

### Statistical analysis

All data were evaluated using Student t-test with SigmaStat statistical software (version 2.0). Statistical significance was defined by P ≤ 0.05 (*) and P ≤ 0.005 (**). All error bars shown in figures and those quoted following the +/- signs represent standard deviation.

## Results

### Activation of Cdc42 by C. jejuni is time-dependent, and bacterial invasion requires intact lipid rafts and Cdc42 expression

**Figure 1:**
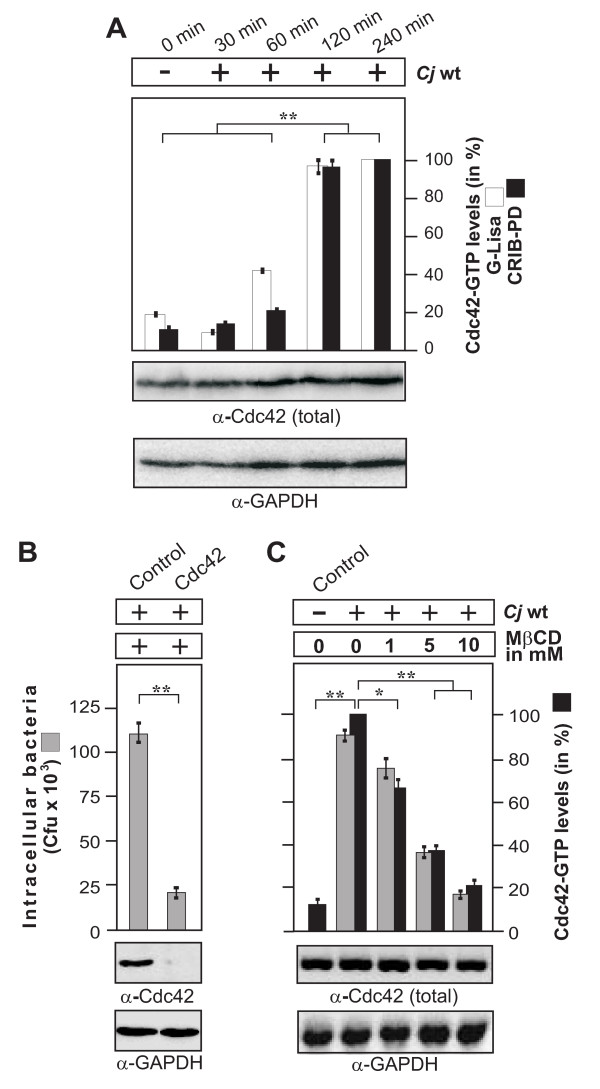
***C. jejuni*-triggered Cdc42 activation is time-dependent and requires intact lipid rafts**. (A) Quantification of Cdc42 activity during the course of infection. INT-407 cells were infected with wt *C. jejuni *81-176 for indicated periods of time. The presence of active Cdc42-GTP was quantified by G-Lisa and GST-CRIB pulldown. One hundred % of GTPase activity corresponds to the highest amount of detected Cdc42-GTP level (right lane). Similar quantities of total Cdc42 and GAPDH were confirmed by Western blotting. (B) Effect of Cdc42 expression knockdown on *C. jejuni *invasion. INT-407 cells were transfected with Cdc42-siRNA as well as a scrambled siRNA as control. After 48 hours, cells were infected with *C. jejuni *for 6 hours. Intracellular bacteria were quantified by gentamicin protection assays. Immunoblotting with α-Cdc42 antibody confirmed down-regulation of the protein. GAPDH expression levels were determined as control. (C) Effects of MβCD targeting lipid rafts on host cell internalization of *C. jejuni*. INT-407 monolayers were pre-incubated with the indicated concentrations of MβCD for 30 min, followed by 6 hours infection with wt *C. jejuni *84-25. Intracellular *C. jejuni *were quantified by gentamicin protection assays. The presence of active Cdc42-GTP was analyzed by CRIB-GST pulldown and quantified. One hundred % of activity corresponds to the highest amount of detected Cdc42-GTP level (lane 2). Similar quantities of total Cdc42 and GAPDH were confirmed by Western blotting. (*) P ≤ 0.05 and (**) P ≤ 0.005 were considered as statistically significant as compared to the control.

We have previously shown that small Rho GTPases such as Cdc42 are activated by *C. jejuni*. Inhibitors, toxins, expression of dominant-negative constructs and other experiments have indicated that active Cdc42 could be an important host determinant required for bacterial invasion [20]. In the present study, we identified and characterized the signaling pathway leading to *C. jejuni*-induced Cdc42 activation. First, we confirmed that Cdc42 is activated in infected non-phagocytic INT-407 intestinal epithelial cells using a novel commercial G-Lisa assay and GTPase pulldowns of the GST-CRIB construct. The results showed that infection with wild-type (wt) *C. jejuni *strains 81-176, F38011 or 84-25 induced the accumulation of active Cdc42-GTP in a time dependent manner (Figure 1A and data not shown). In order to confirm that Cdc42 is indeed necessary for the entry of *C. jejuni *into host cells, we downregulated Cdc42 expression by siRNA. Downregulation of Cdc42 expression by > 95% lead to a significant drop in the number of intracellular colony-forming units (CFU), as quantified in gentamicin protection assays (Figure 1B). Transfection of a scrambled siRNA as control did not reveal suppressive effects on *C. jejuni *invasion (Figure 1B). These results indicate that invasion of *C*. *jejuni *into cultured host cells requires Cdc42.

Recent experiments have indicated that treatment with methyl-beta cyclodextrin (MβCD), an agent sequestering cholesterol in lipid rafts, decreased the ability of *C. jejuni *to invade cultured epithelial cell lines [30]. Thus, we tested if the integrity of lipid rafts may be also required for *C. jejuni*-mediated Cdc42 activation. Indeed, addition of MβCD to INT-407 cells inhibited *C. jejuni*-induced Cdc42 activation and bacterial internalization in a dose-dependent fashion (Figure 1C), suggesting that one or more lipid raft-associated host cell receptor(s) maybe activated by the bacteria to induce signaling resulting in elevated Cdc42-GTP levels and subsequently bacterial uptake.

### C. jejuni invasion and Cdc42 activation require fibronectin, integrin-β1, FAK and Src kinases

**Figure 2:**
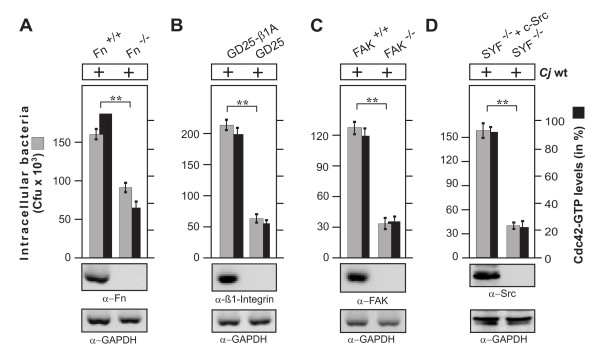
**Importance of fibronectin, integrin-β1, FAK and Src kinases expression on *C. jejuni *invasion**. The following cells lines were infected with wt *C. jejuni *81-176 for 6 hours. (A) Fibronectin-deficient cells (Fn^-/-^) and corresponding floxed wt cells (Fn^+/+^), (B) integrin-β1-deficient cells (GD25) and GD25 stably re-expressing wt integrin-β1A (GD25-β1A) cells, (C) FAK-deficient cells (FAK^-/-^) and FAK^-/- ^cells stably re-expressing wt FAK and (D) Src kinase-deficient cells (SYF^-/-^) and SYF^-/- ^cells stably re-expressing wt c*-src*. Intracellular *C. jejuni *were quantified by gentamicin protection assays, and Cdc42 activation by CRIB-GST pulldowns. (**) P ≤ 0.005 was considered as statistically significant. Fibronectin, integrin-β1, FAK and Src expression was analyzed by immunoblotting. GAPDH expression levels were determined as loading control.

Because *C. jejuni *encodes the well-known fibronectin-binding protein CadF on its surface [19], we suggested that a classical fibronectin→integrin-β1→focal adhesion kinase (FAK)→Src kinase pathway could be involved in activating Cdc42. To investigate this hypothesis, we used fibroblast cell lines derived from fibronectin^-/-^, integrin-β1^-/- ^(so called GD25 cells), FAK^-/- ^and c*-src*^-/-^, c*-yes*^-/-^, and c*-fyn*^-/- ^(SYF) triple knockout mice [36,38,41,42], which completely lack expression of the respective genes (Figure 2A-D). As positive control, we infected with wt *C. jejuni *under identical conditions floxed fibronectin^+/+ ^cells, GD25 cells re-expressing wt integrin-β1A (GD25β1A), FAK^-/- ^cells re-expressing wt FAK and SYF cells re-expressing c-Src. Gentamicin protection assays showed that, while the knockout cells exhibited significant deficiencies for bacterial uptake, *C. jejuni *invaded all wt control cells very efficiently (Figure 2A-D). In addition, Cdc42-GTP levels were determined in the same set of experiments, showing that activation of Cdc42 also depends on the expression of each of the above genes.

### C. jejuni invasion is inhibited in cells expressing integrin-β1 mutants with defects in fibronectin fibril formation and FAK signaling

**Figure 3:**
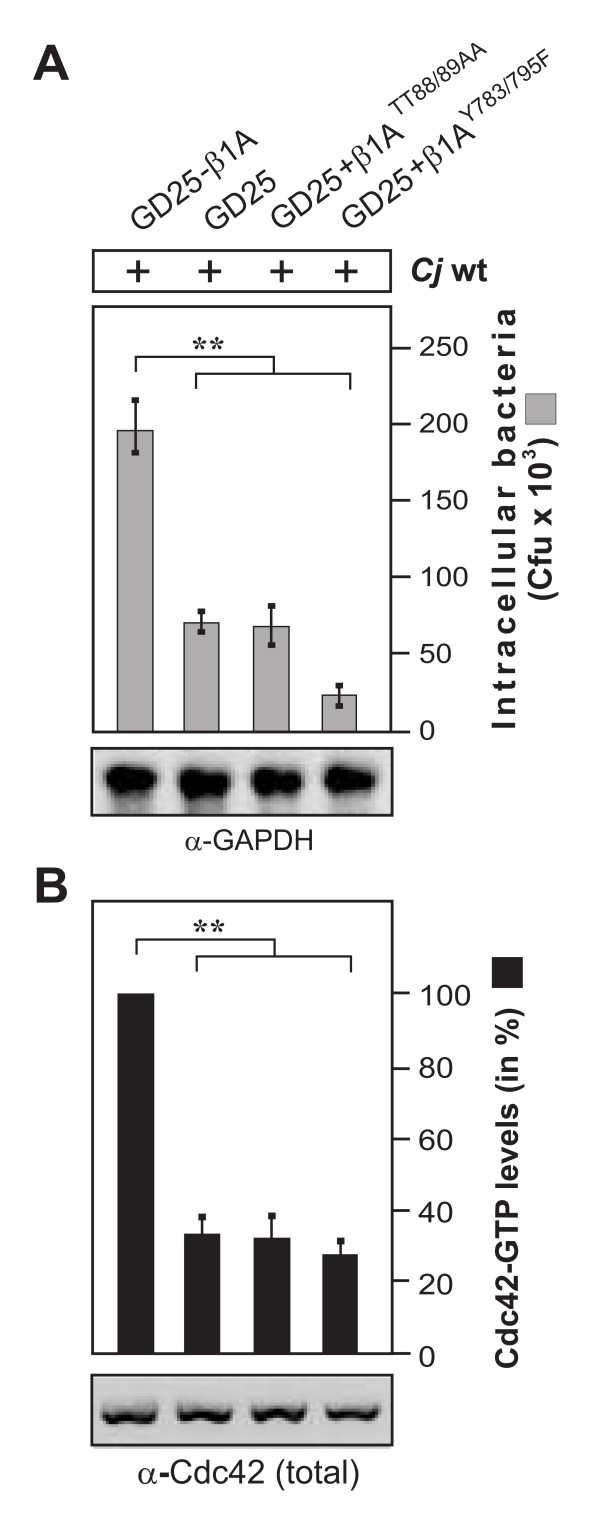
***C. jejuni *invasion is impaired in cells expressing integrin-β1 point mutations exhibiting defects in fibronectin fibril organisation or FAK phosphorylation**. (A) Integrin-β1-deficient cells (GD25) and GD25 stably re-expressing mutated integrin subunit β1A (GD25-β1 ATT788-9AA or GD25-β1 AY783/795F or wild-type β1A (GD25-β1A) cells were infected with wt *C. jejuni *81-176 for 6 hours. Intracellular *C. jejuni *were quantified by gentamicin protection assays. (B) The presence of active Cdc42-GTP was quantified by CRIB-GST pulldowns. One hundred % of activity corresponds to the highest amount of detected Cdc42-GTP level. (**) P ≤ 0.005 was considered as statistically significant. Similar quantities of total Cdc42 and GAPDH were confirmed by Western blotting.

In order to investigate the importance of fibronectin and integrin-β1 signaling in more detail, we utilized two well-established mutant cell lines, GD25 knockout cells stably expressing integrin-β1A TT788/89AA which exhibit functional FAK signaling but a defect in extracellular fibronectin fibril formation [39], and GD25β1A-Y783/795F cells, which have a pronounced defect in FAK autophosphorylation at Y-397 [40]. These cells were infected with *C. jejuni *followed by gentamicin protection assays. The number of intracellular *C. jejuni *was found to be significantly reduced in the integrin-β1-deficient GD25 cells and was restored when wt integrin-β1A was stably expressed (Figure 3A). However, the expression of TT788/89AA or Y783/795F mutants in GD25 cells did not rescue the capability of *C. jejuni *to invade these cells; especially the FAK-signaling deficient GD25β1A-Y783/795F cells exhibited a highly pronounced defect for the uptake of bacteria (Figure 3A). Interestingly, these results strictly correlated with the parallel Cdc42 activation assays, showing that the same integrin-β1 mutant cell lines are also widely deficient in their capability to induce Cdc42-GTP production during *C. jejuni *infection (Figure 3B). This data suggests that integrin-mediated fibronectin fibril formation and FAK downstream signaling are also required for efficient Cdc42 activation and *C. jejuni *uptake.

### C. jejuni induces filopodia formation and invasion in wt cells but not in fibronectin, integrin-β1 and FAK knockout cells

**Figure 4:**
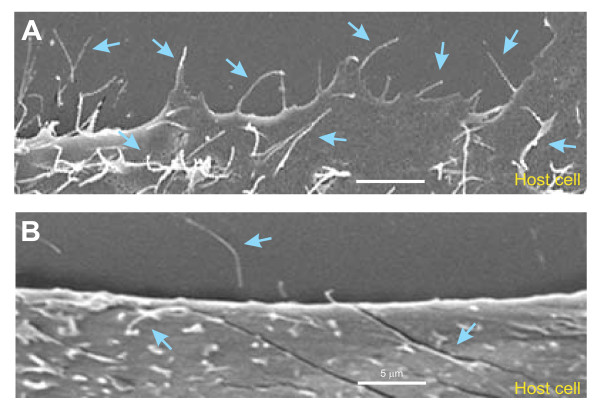
**High resolution FESEM of *C. jejuni-*induced filopodia formation**. Representative sections of wild-type fibroblasts incubated for 6 hours with wt *C. jejuni *81-176 (A) and non-infected fibroblast control cells that were mock-treated (B) are shown. Infection revealed the occurrence of membrane protrusion events with long filopodia at the periphery and on top of cells which were only sporadically seen in the non-infected control cells (blue arrows).

**Figure 5:**
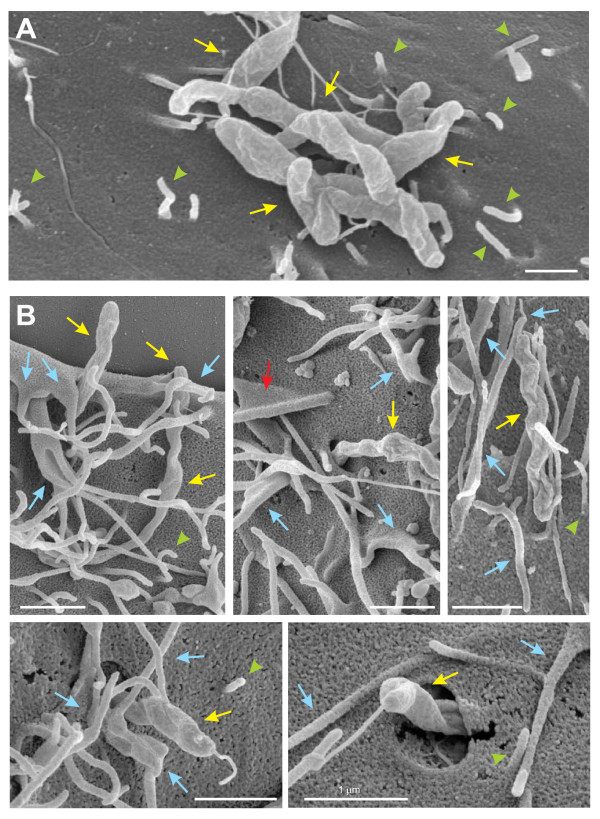
**High resolution FESEM of *C. jejuni-*induced filopodia formation and invasion**. (A) Infection of GD25 knockout cells with wt *C. jejuni *81-176 (yellow arrows) for 6 hours revealed bacterial attachment to the cell surface with short microspikes (green arrowheads) present, but membrane dynamics events or invasion were rarely seen. Similar observations were made with infected fibronectin^-/- ^or FAK^-/- ^cells. (B) Infecting *C. jejuni *in wt cells were regularly associated with long filopodia (blue arrows) membrane ruffling (red arrows), as well as elongated microspikes (green arrowheads).

The above results led us to propose that fibronectin, integrin-β1 and FAK may form a signaling complex to induce Cdc42 activity during infection. Thus, we asked if we could visualize classical Cdc42-triggered filopodia on cells upon contact with the bacteria. To investigate this question, we infected wt fibroblasts with wt *C. jejuni *followed by analysis of host cells by FESEM. The micrographs showed that *C. jejuni *profoundly induced filopodia formation at the periphery and top of infected host cells (Figure 4A, up to 7 μm long, blue arrows), while only very few of these structures could be seen in non-infected control wt fibroblasts (Figure 4B). Next, we infected fibronectin^-/-^, GD25 and FAK^-/-^knockout cell lines and their corresponding wt control cells, followed by the analysis of the interaction of *C. jejuni *with the surface of host cell surface by high-resolution FESEM. Infection of fibronectin^-/-^, GD25 and FAK^-/-^knockout cell lines revealed the presence of attached bacteria (yellow arrows) at the surface of the cells with short microspikes (up to 1 μm long, green arrowheads), but no indication of induced membrane dynamics was seen (Figure 5A). Filopodia formation or invading *C. jejuni *could be detected only rarely in any infected knockout cell line. In contrast, infection of wt fibroblasts under the same conditions revealed tight engulfment of the attached bacteria associated with long filopodia (blue arrows) and/or ruffles (red arrows) and somewhat elongated microspikes (green arrowheads) as shown in Figure 5B. In agreement with our earlier observation in infected INT-407 cells [20], we found that *C. jejuni *entered the wt fibroblasts in a very specific fashion, first with its flagellum followed by the bacterial cell with the opposite flagellar end (Figure 5B, bottom). The generation of filopodia in wt cells confirms the typical occurrence of Cdc42 GTPase activation during infection, followed by dynamic membrane rearrangements and host entry, dependent on the expression of fibronectin, integrin-β1 and FAK.

### Wild-type but not ΔcadF mutant C. jejuni induces profound FAK, EGFR and PDGFR phosphorylation

**Figure 6:**
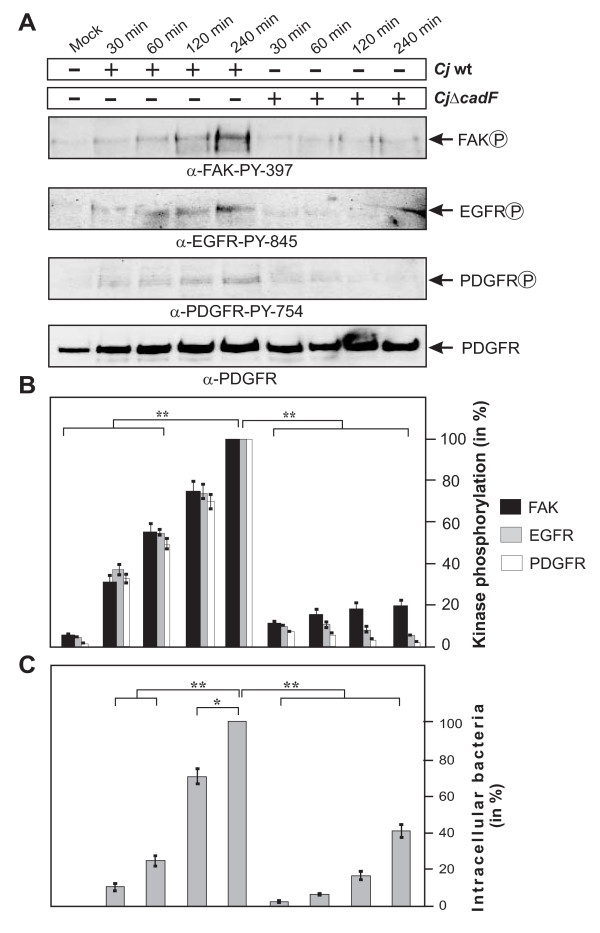
**Importance of CadF for *C. jejuni-*induced FAK, EGFR and PDGFR activation**. (A) FAK-positive fibroblasts were infected with wt *C. jejuni *F38011 or isogenic F38011Δ*cadF *for indicated periods of time. FAK, EGFR or PDGFR activation was analysed by immunoblotting with indicated antibodies. Total PDGFR expression levels were determined as loading control. (B) Quantification of FAK, EGFR and PDGFR kinase phosphorylation during the course of infection. One hundred % of activity corresponds to the highest amount of phosphorylation detected per experiment and selected kinase (lane 5). (C) Intracellular *C. jejuni *were quantified by gentamicin protection assays. (*) P ≤ 0.005 and (**) P ≤ 0.005 were considered as statistically significant.

Next, we aimed to investigate if infection activates FAK autophosphorylation and if this is associated with the activation of EGFR and PDGFR receptors, which are also present in membrane lipid rafts. We therefore infected host cells with wt *C. jejuni *and an isogenic Δ*cadF *deletion mutant in a time course. Protein lysates from the infected cells were prepared and subjected to Western blotting using activation-specific phospho-antibodies for FAK, EGFR and PDGFR (Figure 6A). The results show that wt *C. jejuni *significantly induced the autophosphorylation of FAK at tyrosine residue Y-397 in the active centre, the phosphorylation of EGFR at Y-845 and the phosphorylation of PDGFR at Y-754 over time (Figure 6A). The data indicated that maximal levels of kinase activity appeared after 4 hours of infection (Figure 6B), which correlated with increasing Cdc42-GTP levels over time (Figure 1A) and the invasion capabilities of wt *C. jejuni*, as determined by gentamicin protection assays (Figure 6C). Interestingly, infection with the Δ*cadF *mutant, as examined under identical conditions, revealed that phosphorylation of FAK, EGFR and PDGFR were widely impaired (Figure 6A, B) and correlated with the reduced invasiveness of this mutant (Figure 6C). These observations suggest that CadF maybe involved in *C. jejuni-*induced FAK, EGFR and PDGFR kinase activities, and host cell invasion.

### Induction of maximal Cdc42-GTP levels requires CadF and is strongly impaired in FAK^-/- ^knockout cells

**Figure 7:**
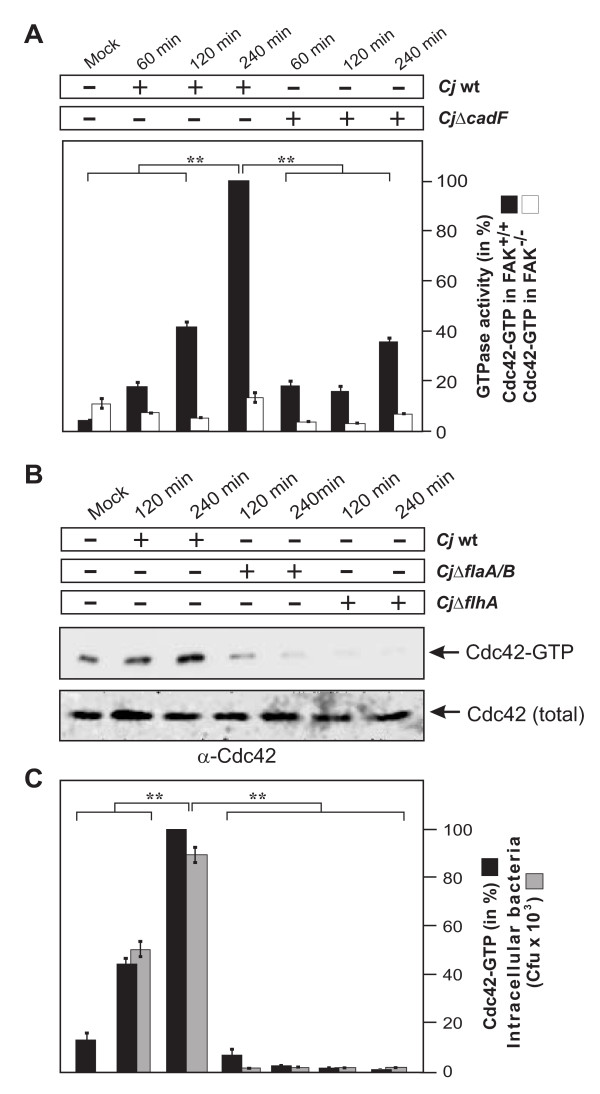
**Importance of the CadF and flagellar apparatus for *C. jejuni*-induced activation of Cdc42 and bacterial invasion**. (A) FAK^+/+ ^and FAK^-/- ^cells were infected with wt *C. jejuni *F38011 or isogenic F38011Δ*cadF *for the indicated periods of time. Quantification of Cdc42-GTP levels by CRIB-GST pulldown during the course of infection. One hundred % of activity corresponds to the highest amount of detected Cdc42-GTP level (lane 4). **(**B) FAK-positive cells were infected with the indicated strains in a time-course. The presence of bound, active Cdc42-GTP was analyzed in CRIB-GST pulldown assays followed by Western blotting using α-Cdc42 antibody. Similar quantities of individual GTPases at every time point were confirmed by Western blotting using equivalent volumes of cell lysates. (C) Quantification of Cdc42-GTP levels during the course of infection. One hundred % of activity corresponds to the highest amount of detected Cdc42-GTP level (lane 3). The amount of intracellular bacteria was quantified by gentamicin protection assays under the same experimental conditions. (**) P ≤ 0.005 were considered as statistically significant as compared to the control.

To investigate if FAK is required for *C. jejuni-*induced Cdc42 activation, we infected FAK^-/- ^knockout cells and cells re-expressing FAK under the same conditions with wt *C. jejuni *and Δ*cadF *mutant, followed by CRIB-GST pulldown assays. While growing levels of activated Cdc42 were detected in FAK-positive cells over time with wt *C. jejuni*, no detectable activation of Cdc42 was found in FAK^-/- ^cells during the entire course of infection (Figure 7A). This suggests that FAK is involved in signaling upstream of Cdc42 activation during invasion of *C. jejuni*. Furthermore, significantly reduced Cdc42-GTP levels were observed in both FAK-positive and FAK^-/- ^cells infected with the Δ*cadF *mutant (Figure 7A). These findings further support the notion that CadF could be a significant player in signaling leading to FAK-mediated activation of Cdc42. However, the Δ*cadF *mutant was still able to induce some Cdc42 GTPase activation in FAK-positive cells, suggesting that other bacterial factor(s) are also implicated in this signaling cascade (Figure 7A).

### The C. jejuni flagellum is also involved in Cdc42 activation and bacterial invasion

Because CadF is not the sole bacterial factor involved in *C. jejuni*-induced Cdc42 activity, we searched for other bacterial factors involved in this signaling. The *C. jejuni *flagellar apparatus has been reported to be a major pathogenicity determinant [25,26,48]. To test if an intact flagellum plays a role in *C. jejuni*-induced Cdc42 activation, host cells were infected with wt strain 81-176 and its isogenic mutants Δ*fla*A/B lacking the two major flagellar subunits FlaA and FlaB [35], and Δ*flhA*, a key element involved in the regulation of flagellar genes and other pathogenicity factors in *C. jejuni *[49]. As expected, activated Cdc42 was detected in FAK-positive cells between 2-4 hours after infection with wt *C. jejuni *(Figure 7B, C). In contrast, no detectable Cdc42 activation and host cell invasion was found in cells infected with Δ*fla*A/B or Δ*flhA *mutants during the entire course of infection (Figure 7B, C). This indicates that, in addition to the contribution by CadF as shown above, the intact *C. jejuni *flagellum may also play a role in the activation of Cdc42.

### The guanine exchange factor Vav2 is required for C. jejuni-mediated Cdc42 activation

**Figure 8:**
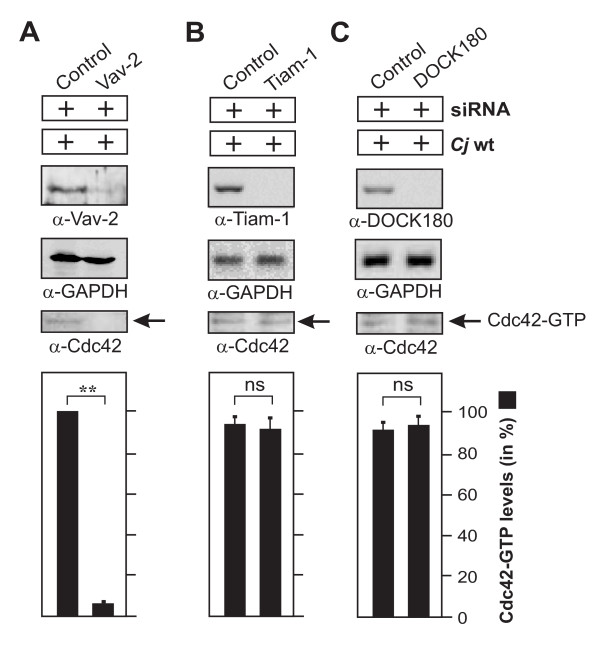
**Importance of guanine exchange factor Vav2 for *C. jejuni*-induced Cdc42 activation**. INT-407 cells were transfected for 48 hours with siRNA for Vav2 (A), Tiam-1 (B) or DOCK180 (C) as well as a scrambled siRNA as control. Immunoblotting with the indicated antibodies confirmed knockdown of the respective proteins. GAPDH expression levels were determined as control. Quantification of Cdc42 GTPase activity after infection with wt *C. jejuni *81-176 for 6 hours. The presence of bound, active Cdc42-GTP was analyzed in CRIB-GST pulldown assays followed by Western blotting using α-Cdc42 antibody. One hundred % of activity corresponds to the highest amount of detected Cdc42-GTP level (lane 1).

The following aim was to determine additional signaling factors downstream of FAK and upstream of Cdc42 activation. Cycling of small Rho GTPases between the inactive and active forms is commonly stimulated by a class of proteins called guanine nucleotide exchange factors (GEFs) and negatively regulated by GTPase activating proteins (GAPs). GEFs trigger the exchange of GDP for GTP to generate the active form of a given GTPase, which is then capable of recognizing downstream targets [50-52]. To identify which GEF(s) is/are involved in *C. jejuni*-induced Cdc42 activation, the expression of typical GEFs including Vav2, DOCK180 or Tiam-1 was downregulated using target-specific siRNA, followed by infection and CRIB-GST pulldowns. While the downregulation of Vav2 led to the predominant inhibition of Cdc42-GTP levels (Figure 8A), both downregulation of Tiam-1 and DOCK180 (Figure 8B, C) or transfection of non-targeting scrambled siRNA control had no significant effect on *C. jejuni*-triggered Cdc42-GTP production (Figure 8A-C). These findings suggest that Vav2, but not Tiam-1 or DOCK180, plays a crucial role in *C. jejuni-*induced Cdc42 activation.

### Vav2 is required for maximal host cell invasion by C. jejuni

**Figure 9:**
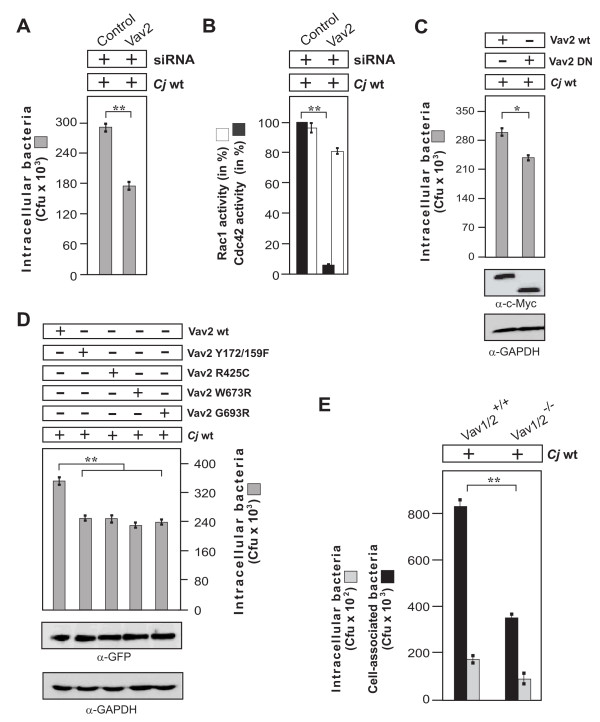
**Downregulation, elimination or **i**nterference with important Vav2 functions reduces the uptake of *C. jejuni *in host cells**. (A) INT-407 cells were transfected with siRNA against Vav2 or a scrambled siRNA as control. After 48 hours, cells were infected with wt *C. jejuni *81-176 for 6 hours. Intracellular bacteria were quantified by gentamicin protection assays. (B) The presence of active Rac1-GTP and Cdc42-GTP was quantified by CRIB-GST pulldowns. One hundred % of activity corresponds to the highest amount of detected GTPase-GTP level. (C) INT-407 cells were transfected with indicated Myc-tagged or (D) GFP-tagged Vav2 constructs. After 48 hours, cells were infected with wt *C. jejuni *81-176 for 6 hours. Intracellular bacteria were quantified by gentamicin protection assays. Expression of the individual Vav2 constructs was verified by Western blot analysis. GAPDH expression levels were determined as control. (E) Vav2-deficient cells (Vav1/2^-/-^) or Vav2-expressing control fibroblasts (Vav1/2^+/+^) were infected for 6 hours with *C. jejuni*. Intracellular and cell-associated bacteria were quantified by gentamicin protection assays. (*) P ≤ 0.05 and (**) P ≤ 0.005 were considered as statistically significant.

Next we aimed to consolidate our understanding of the potential importance and role of Vav2 during infection. First, to identify if Vav2 is also involved in host cell invasion by *C. jejuni*, the expression of Vav2 was suppressed with siRNA, followed by infection and gentamicin protection assays. The results showed that downregulation of Vav2 led to a significant drop in the amount of intracellular bacteria (Figure 9A). In addition, we investigated if the downregulation of Vav2 may influence the activity of another small Rho GTPase, Rac1. Quantification of GTPase activation levels indicated that while a significant suppressive effect was seen on Cdc42-GTP, only a slight reduction of Rac1-GTP levels were observed (Figure 9B). This suggests that Vav2 may predominantly target Cdc42 in infected INT-407 cells. Further evidence for an important function of Vav2 in host cell invasion came from the use of dominant-negative Vav2. Expression of dominant-negative Myc-tagged Vav2, but not wt Myc-tagged Vav2, also had some downregulatory effect on *C. jejuni *invasion (Figure 9C).

### Signaling of Vav2 is functionally linked to growth factor receptors EGFR and PDGFR

As siRNA-mediated gene silencing or expression of dominant-negative Vav2 interfered with uptake of *C. jejuni*, the impact of Vav2 on *C. jejuni *host cell entry was examined in more detail. Vav2 is a substrate of EGFR/PDGFR kinases and GTPases including Cdc42 can be activated downstream of both receptors through Vav2 exchange activity [46,53-55]. For this purpose, INT-407 cells were transiently transfected with wt Vav2 and different Vav2 mutants that were either impaired in EGFR-dependent phosphorylation of Vav2 (Vav2 Y172/159F), lacked the primary phosphatidylinositol 3, 4, 5-triphosphate binding site (Vav2 R425C) or were not capable of binding to activated EGFR (Vav2 W673R and Vav2 G693R) [46]. Gentamicin protection assays revealed that overexpression of either Vav2 mutant construct significantly reduced the number of intracellular *C. jejuni *bacteria (Figure 9D), further confirming the importance of Vav2 in bacterial uptake. These findings also support the view that Vav2, by binding to and signaling through a *C. jejuni-*induced EGFR/PDGFR and PI3-K kinase activation pathway, may contribute Cdc42 activation during infection. Finally, we utilised Vav1/2^-/- ^knockout fibroblasts for infection and gentamicin protection assays. The determination of total cell-associated and intracellular *C. jejuni *bacteria in the same set of experiments showed that expression of Vav is not only important for invasion but has also a significant effect on the binding of *C. jejuni *to these cells (Figure 9E).

### The activities of FAK, EGFR, PDGFR and PI3-K are also important for C. jejuni-induced Cdc42-GTP levels and invasion

**Figure 10:**
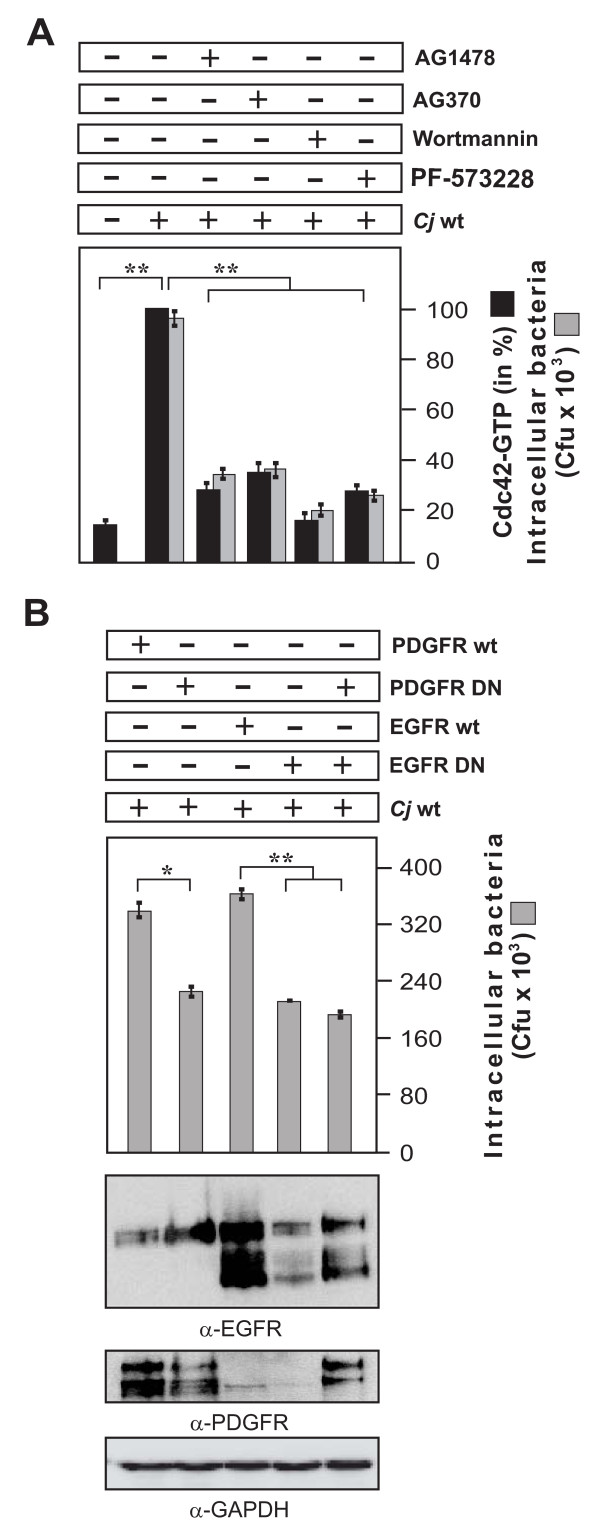
**Importance of FAK, EGFR, PDGFR and PI3-kinase activities for *C. jejuni*-induced activation of Cdc42 and bacterial invasion**. (A) INT-407 monolayers were pre-incubated for 30 min with the indicated pharmacological inhibitors and infected with *C. jejuni *for 6 hours. Intracellular *C. jejuni *were quantified by gentamicin protection assays. The presence of active Cdc42-GTP was quantified by CRIB-GST pulldowns. One hundred % of activity corresponds to the highest amount of detected Cdc42-GTP level (lane 2). (B) Effect of overexpression of dominant-negative forms of PDGFR and EGFR on *C. jejuni *uptake. 48 hours post transfection INT-407 cells were infected with *C. jejuni *for 6 hours. Intracellular bacteria were quantified by gentamicin protection assays. Expression of the individual constructs was verified by Western blotting. GAPDH expression levels were determined as control. (*) P ≤ 0.05 and (**) P ≤ 0.005 were considered as statistically significant.

Finally, we wanted to investigate if pharmacological inhibition of the above described host cell kinases could confirm the proposed signaling pathway leading to Cdc42 activation and *C. jejuni *invasion. For this purpose, INT-407 cells were pre-treated for 30 min with AG1478 (EGFR inhibitor), AG370 (PDGFR inhibitor), wortmannin (PI3-K inhibitor) or PF-573228 (FAK inhibitor) followed by infection with wt *C. jejuni*. The results showed that inhibition of each of these kinases had a profound suppressive effect on both Cdc42-GTP levels and bacterial invasion (Figure 10A). To further corroborate these findings, INT-407 cells were transiently transfected with wt PDGFR and EGFR constructs, and their respective dominant-negative forms, followed by infection with *C. jejuni*. Gentamicin protection assays showed that overexpression of either dominant-negative mutant also significantly reduced the amount of recovered intracellular *C. jejuni*, confirming the involvement of PDGFR and EGFR in uptake of *C. jejuni *(Figure 10B). These data collectively suggest that we have identified a novel important pathway of *C. jejuni *host cell entry, proceeding by the activation of a fibronectin→integrin-beta1→FAK/Src→EGFR/PDGFR→PI3-kinase→Vav2→Cdc42 signaling cascade.

## Discussion

**Figure 11:**
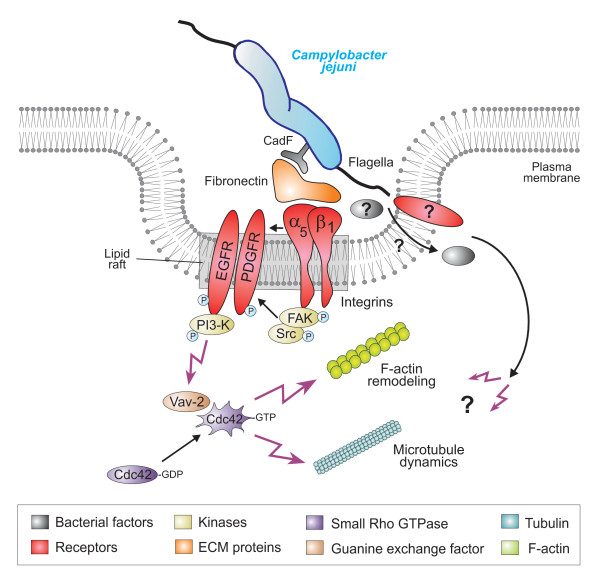
**Model for *C. jejuni*-induced signaling leading to Cdc42 activation and bacterial invasion**. *C. jejuni *adheres to host cells *via *the fibronectin-binding protein CadF, which acts as a bridge engaging the integrin-β1 receptor. Integrin occupancy and clustering in lipid rafts leads to recruitment and activation of the non-receptor tyrosine kinase FAK. Phosphorylation of FAK and Src triggers a cascade of signals resulting in the formation of protein complexes leading to activation of other signaling factors as indicated. Assembly of integrin-dependent signal complexes leads to phosphorylation and transactivation of PDGFR and EGFR, followed by stimulation of PI3-K and Vav2. Activated Vav2 then induces the activation of Cdc42. This signaling potentially causes localized actin and/or microtubule rearrangements at the site of *C. jejuni *entry, resulting in bacterial uptake. In addition to CadF, the *C. jejuni *flagellum also appears to play a role in the described signal cascades. If the flagellum participates by sole bacterial motility, by translocating bacterial effector proteins or targeting a host receptor directly is not yet clear and needs to be investigated in future studies.

Invasion of host target cells is a major strategy of a large group of pathogenic microbes. The entry process comprises numerous specific steps at the host-pathogen interface including bacterial binding to one or more receptors, delivery of signals to the host cell, re-programming of intracellular host signaling cascades, membrane and cytoskeletal dynamics, followed by engulfment and uptake of the bacterium. These processes commonly involve the activation of small Rho family GTPases. Prominent members are the GTP-binding proteins RhoA, Cdc42 and Rac1, which act as guanine nucleotide-regulated switches to induce various responses during the infection process [50,56-58]. Host cell invasion by the gastrointestinal pathogen *C. jejuni *has been reported to cause substantial tissue damage, but the molecular mechanisms involved remained widely unknown. We could recently demonstrate that *C. jejuni *invasion of INT-407 cells is time-dependent and associated with increasing activities of small Rho GTPases, one of which is Cdc42 [20]. The application of pharmacological inhibitors, GTPase-modifying toxins and expression of constitutive-active or dominant-negative Cdc42 plasmids provided evidence that Cdc42 activity plays a role in host cell invasion of *C. jejuni *[20]. In the present report, we aimed to unravel the cascade of signaling events resulting in *C. jejuni*-triggered Cdc42 activity. Using knockout cell lines of several host receptors (fibronectin^-/-^, GD25 integrin-β1^-/-^) and kinases (FAK^-/- ^and SYF), siRNA transfection, dominant-negative and other expression constructs, G-Lisa, CRIB pulldowns, gentamicin protection assays and electron microscopy, we show that *C. jejuni *exploits a fibronectin→integrin-β1→FAK/Src→EGFR/PDGFR→PI3-kinase→Vav2 signaling pathway, which is crucial for activating Cdc42 GTPase function, involved in invasion of host target cells. Our major findings in this study are discussed below and have been summarised in a signaling model (Figure 11).

The use of specific knockout cell lines for *C. jejuni *invasion-associated signaling studies has the great advantage over other cell systems that clear conclusions can be drawn if the deleted gene of interest is involved in this process or not. Host cell entry of *C. jejuni *was largely reduced in each of the above knockout cell lines, suggesting that fibronectin, integrin-β1, FAK and Src kinases play a crucial role in invasion. Since *C. jejuni *strains express the conserved major fibronectin-binding protein CadF [15,17,18,20] and because fibronectin is the natural ligand for integrin-β1 receptor [59,60], our current findings indicate a cascade of fibronectin→integrin-β1→FAK/Src-dependent signaling events occurring during infection. In line with these observations, we found that Cdc42-GTP levels triggered by *C. jejuni *infection were strongly elevated in cells expressing wt FAK but not in FAK-knockout cells, and Cdc42-GTP upregulation was verified by two independent molecular techniques including GST-CRIB pulldown and G-Lisa. These findings were further supported by the detection of filopodia formation, membrane dynamics and engulfment of *C. jejuni *during infection of wt control cells, but this was widely impaired in any of the infected knockout cell lines. These novel data provide a clear proof that fibronectin, integrin-β1, FAK and Src kinases are crucial host factors playing significant roles in *C. jejuni*-induced Cdc42 activation and filopodia formation, linked to invasion. Thus, by a strategy engaging fibronectin, integrin-β1, FAK and Src, the bacteria appear to hijack the capacity of the integrin receptor complex to connect with the intracellular cytoskeleton and to create the necessary pulling forces to trigger *C. jejuni *entry into host cells.

Integrin-β1-dependent fibrillar cell adhesion in healthy tissues play a crucial role in the organisation of the ECM because they co-align with proper extracellular fibronectin fibril structures [60,61]. Genetic elimination of integrin-β1 in GD25 cells results in profound assembly defects within the fibrillar ECM meshwork including fibronectin [38,60,62]. Cellular pulling forces generated by integrin-β1-mediated linkage to the actin-myosin network therefore appear to be critical for ECM fibronectin fibril formation, as force-triggered conformational changes are essential to expose cryptic oligomerisation motifs within individual fibronectin proteins [60,63]. Importantly, an integrin-β1 TT788/789AA mutant is defective in mediating proper cell attachment and is unable to induce fibronectin fibril formation [39]. The conformation of the extracellular integrin-β1 domain is shifted towards an inactive state but the cytoplasmic part remains functional with respect to activation of FAK. Interestingly, *C. jejuni *was widely unable to enter GD25 cells stably transfected with this integrin-β1 mutant. Therefore, we conclude that threonine residues 788-789, which are of critical importance for integrin-β1 function due to effects on the extracellular conformation and function of the receptor, play also a crucial role in proper for fibronectin fibril organisation, important for efficient *C. jejuni *host cell entry.

Integrin activation and clustering is tightly associated with the activation of FAK, and is a strategy of regulating outside-in signal transduction events leading to cytoskeletal rearrangements [64,65]. Indeed, the lowest numbers of intracellular *C. jejuni *were observed with GD25-β1A-Y783/795F cells which are impaired in signaling to FAK due to a defect in β1-dependent autophosphorylation of FAK at tyrosine residue Y-397 [40]. Despite the defect in integrin-β1-mediated FAK activation, FAK was still localized to focal adhesions. This result suggests that besides signaling of integrin-β1 to form correct fibronectin fibril formation, β1-dependent signaling to FAK activation is also required for *C. jejuni-*induced Cdc42 signaling and bacterial uptake. Indeed, FAK autophosphorylation is strongly activated by *C. jejuni *and pharmacological inhibition of FAK as well as infection of FAK^-/- ^cells did not lead to stimulation of Cdc42 GTPase activity. In addition, FAK^-/- ^mouse embryos *in vivo *as well as *in vitro *cultured FAK^-/- ^cells fail to properly assemble fibronectin fibrils [60,66]. Therefore, the observed deficiency of FAK^-/- ^cells to internalise *C. jejuni *is associated with two phenotypes, inhibited signaling to proper ECM organisation and downstream signaling leading to GTPase activation. Thus, fibronectin/integrin-linkages to the dynamic actin-myosin or microtubuli networks are disrupted in FAK-deficient cells and necessary pulling forces are not provided. This setting is similar to that shown for fibronectin-binding protein-expressing *Staphylococcus aureus*, because infected FAK^-/- ^or fibronectin^-/- ^cells were similarly impaired to internalise these bacteria [37,67]. In addition, the importance of FAK activition has been reported for other pathogens targeting integrins for bacterial invasion or other purposes, including *Yersinia pseudotuberculosis *[68,69], group B Streptococci [70] and *Helicobacter pylori *[71-73]. Thus, FAK appears to be a very common target of multiple bacterial pathogens.

Our observation that FAK activation is required for *C. jejuni*-induced Cdc42 activity and host cell entry, led us to search for other downstream signaling determinants. Using siRNA knockdown, we tested the importance of a few well-known GEFs, including Tiam-1, DOCK180 or Vav2, for the production of Cdc42-GTP levels in infected cells. Interestingly, Vav2 (but not Tiam-1 or DOCK180) was required for *C. jejuni-*induced Cdc42 activation. The importance of Vav2 was then confirmed by the expression of dominant-negative constructs and the use of Vav1/2 knockout cells in infection assays. Bacterial adhesion was also reduced in Vav1/2 knockout cells, which can be explained by reduced GTPase activation as compared to wt cells. This is in agreement with reports showing that Vav2 is also involved in the uptake of other pathogens including *Yersinia *and *Chlamydia *[74,75]. Moreover, in our studies the expression of various point mutations in Vav2 linked the signaling directly to growth factor receptors and PI3-K. The application of selective inhibitors during *C. jejuni *infection showed then that the kinase activities of EGFR, PDGFR and PI3-K are also required for Cdc42 activation. This was also confirmed by the expression of dominant-negative versions of EGFR or PDGFR, which exhibited suppressive effects on *C. jejuni *uptake. Extensive research on the regulation of growth factor receptor activation and signaling by integrin-mediated cell adhesion indicates that these two classes of receptors work cooperatively. Several studies showed that integrin ligation allows for the maximal activation of EGFR or PDGFR, thereby producing robust intracellular signals including small Rho GTPase activation [76,77]. These observations are in well agreement with our findings, suggesting that *C. jejuni *activates, *via *fibronectin and integrins, a FAK/Src→EGFR/PDGFR→PI3-kinase→Vav2→Cdc42 signaling pathway. However, transfection with both DN-PDGFR and DN-EGFR constructs resulted in no additive reduction of *C. jejuni *invasion. These latter finding suggests that besides EGFR and PDGFR other signaling pathway(s) are also implicated in *C. jejuni *internalization.

Our previous study indicated that *C. jejuni *pathogenicity factors such as cytolethal distending toxin CDT, plasmid pVir, the adhesin PEB1 or certain capsular genes are not required for *C. jejuni*-induced Cdc42 activation [20]. We found here that an isogenic Δ*cadF *mutant less efficiently induced activation of Cdc42 as compared to wt *C. jejuni*, suggesting that the fibronectin-binding protein CadF, probably in concert with FlpA [17], could be involved in GTPase activation as shown here for Cdc42. It appears that CadF does not only act as a canonical adhesin for bacterial attachment to fibronectin, but could also stimulate integrins as well as FAK, EGFR and PDGFR kinase activity, which subsequently may activate Vav2 and Cdc42, important for maximal *C. jejuni *invasion. Since Δ*flaA/B *or Δ*flhA *knockout mutants lacking the flagella induced only very little Cdc42-GTP levels, another *C. jejuni *determinant playing a role in Cdc42 activation is the flagellar apparatus. The flagellum appears to be a major colonization determinant of *Campylobacter*, shown to be essential for successful infection of several animal models [78-80]. In addition, FlaA/B proteins play a profound role in *C. jejuni *invasion of epithelial cells [16,81-83]. However, the possible impact of flagellar proteins in host cell entry is controversial in the literature. One hypothesis is that the flagella, like their evolutionary related type-III secretion system counterparts, can secrete invasion-associated factors such as CiaB and others into the culture supernatant [15,17,25,48]. The other hypothesis is that flagella-mediated bacterial motility is the driving force to permit host cell entry, but deletion of *ciaB *has no impact [29]. Thus, it is still not clear if the flagellum, unlike its well-known function in bacterial motility, may transport bacterial effectors into the medium or into the host cell. Alternatively, the flagellum itself may target a host cell receptor directly to trigger Cdc42 signaling involved in invasion, which should be investigated in future studies [Figure 11].

## Conclusion

In summary, we provide here several lines of evidence for a novel invasion-related signaling pathway of *C. jejuni *involving fibronectin, integrin-β1, FAK, Src, EGFR, PDGFR, PI3-K, Vav2 and Cdc42 using three different strains including the fully-sequenced model isolate 81-176. Based on our electron microscopic observations and the use of *C. jejuni *mutants in signaling studies, we propose that the flagellum by providing bacterial motility may bring the CadF adhesin in the right position, but may also have other effects, in order to trigger host cell signaling leading to elevated Cdc42-GTP levels and invasion (Figure 11). Interestingly, it appears that the Cdc42-pathway discovered here is not the sole pathway involved in *C. jejuni *invasion. Our observations support the view that another signaling cascade involves the small Rho GTPase member Rac1 [20], which is activated by a pathway involving the same upstream components (fibronectin, integrin-β1 and FAK) but two other GEFs, DOCK180 and Tiam-1 [84], which are obviously not involved in *C. jejuni*-induced Cdc42 activation as shown here. These findings suggest that *C. jejuni *targets two major Rho GTPases by two independent downstream signal transduction pathways and therefore provide novel aspects to our knowledge on the mechanism of *C. jejuni *host cell entry. In future studies it will be important to investigate the precise mechanism of how active Cdc42 regulates microtubule dynamics and/or actin rearrangements involved in providing the necessary pulling forces crucial for the bacterial invasion process.

## List of abbreviations used

CadF: Campylobacter adhesin to fibronectin; *C. jejuni*: *Campylobacter jejuni*; CiaB: Campylobacter invasion antigen B; CRIB: Cdc42-Rac1 interactive binding; GST-CRIB: domain of kinase PAK1 fused to glutathione S-transferase; CFU: colony forming unit; ECM: extracellular matrix; EGFR: epidermal growth factor receptor; FCS: fetal calf serum; FESEM: field emission scanning electron microscopy; FAK: focal adhesion kinase; FlpA: Fibronectin like protein A; GAP: GTPase activating protein; GEF: Guanine exchange factor; JlpA: Jejuni lipoprotein A; kpsS: capsular gene; MβCD: methyl-beta cyclodextrin; GD25 cells: integrin β1^-/- ^mouse fibroblasts; MH agar: Mueller Hinton agar; MOI: multiplicity of infection; PDGFR: platelet-derived growth factor receptor; PEB1: Periplasmic binding protein 1; PI3-K: phosphatidylinositol 3-kinase; siRNA: silencing RNA; PVDF: polyvinylidenedifluoride; *waaF*: heptosyltransferase II gene; wt: wild-type.

## Competing interests

The authors declare that they have no competing interests.

## Authors' contributions

MKG, MB, MR and NT performed and designed the experiments. ST, LB and OO provided crucial materials and advise for the experiments. SB, the senior/corresponding author, supervised the experiments and wrote the manuscript with the help of LB and OO. All co-authors read and approved the final manuscript.
